# How Sodium Dodecyl Sulfate Micelles Affect the Coordination and Peroxidase‐Like Activity of the Hemin–Aβ16 Complex

**DOI:** 10.1002/cplu.202500304

**Published:** 2025-08-06

**Authors:** Chiara Bacchella, Simone Novellini, Elisa Miotto, Stefania Nicolis, Enrico Monzani, Simone Dell’Acqua

**Affiliations:** ^1^ Dipartimento di Chimica Università di Pavia Via Taramelli 12 27100 Pavia Italy

**Keywords:** alzheimer's disease, amyloid‐β, membrane‐like environment, oxidative stress, redox active metals

## Abstract

Alzheimer's disease is an age‐related neurodegenerative disorder and the main cause of dementia in the elderly. The accumulation of metal ions, including iron‐heme, their interaction with amyloid‐β (Aβ) peptides and their ability to catalyze reactive oxygen species formation significantly contribute to the pathogenesis of the disorder. These factors are highly dependent on the surrounding environment, whether intracellular, extracellular, or membrane‐associated. In this study, the interaction between heme and Aβ within a membrane‐mimicking system using sodium dodecyl sulfate is investigated. UV–vis and circular dichroism data indicate that the heme/Aβ complex can be sequestered within the micelle, leaving part of Aβ largely exposed at the surface. The presence of the micellar environment significantly affects both the stability and aggregation state of the hemin group, as well as its accessibility to peptide coordination and to external molecules, such as phenols and catechols. Indeed, peroxidase‐like activity studies show that the overall reactivity of the hemin–Aβ complex is markedly reduced under these conditions. Overall, these results suggest that a membrane‐like environment may offer partial neuroprotection against heme‐induced toxicity by limiting the formation of the heme–Aβ complex and the oxidative damage to nearby biomolecules, including neurotransmitters.

## Graphical abstract

The interaction between iron‐heme and amyloid‐β (Aβ) peptides can be strongly influenced by the cellular compartment. The presence of sodium dodecyl sulfate as a lipid‐mimicking system shows a protective role of the membrane by hindering both the formation of the heme–Aβ complex and the peroxidase‐like activity, reducing the accessibility of the lipid‐bound catalyst to biological substrates, as neurotransmitters.

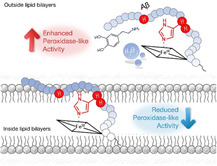

## 1 Introduction

Alzheimer's disease (AD) is a neurodegenerative disorder characterized by a progressive decline in cognitive abilities. The main pathological hallmarks of AD include: 1) the accumulation of amyloid plaques and neurofibrillary tangles, 2) oxidative stress, 3) cholinergic deficits, and 4) neuroinflammation.^[^ ^1^ ^]^ The multifactorial nature of the disease significantly limits the effectiveness of current therapeutic approaches, which primarily offer symptomatic relief rather than a cure.^[^ ^2^ ^]^ However, among the different hypotheses that drive the anti‐AD drug development, the imbalance between free radical production and the brain's antioxidant defense^[^ ^3^ ^,^ ^4^ ^]^ and the impairment in amyloid‐β (Aβ) clearance (leading to its aggregation into insoluble fibrils)^[^ ^5^ ^]^ remain the main targets.

On the other hand, both of these pathological mechanisms are closely connected to the metal dyshomeostasis; as a result, several potential therapeutic agents have been developed with the aim of restoring metal balance in neurons.^[^ ^6^, ^7^ ^–^ ^8^ ^]^

Essential metal ions, such as zinc, copper, and iron (normally involved in neuronal functions) over‐accumulate under AD conditions, thus leading to structural and functional alterations in essential biomolecules, as proteins, lipids, and nucleic acids, ultimately contributing to neuronal damage.^[^ ^9^ ^,^ ^10^ ^]^

Iron, which is the most abundant transition metal in the human body, has also been linked to disease progression through its role as a heme cofactor. Heme has recently been shown to interact specifically with Aβ peptides, contributing to cytotoxic effects in the brain. Several alterations in heme metabolism have been detected in the brains of AD subjects, as well as the colocalization of heme within senile plaques.^[^ ^11^, ^12^, ^13^, ^14^ ^–^ ^15^ ^]^ In fact, in the AD neuronal tissues, higher levels of heme *b* (about 250%) have been observed, and this excess heme content has been linked to an enhancement in reactive oxygen species (ROS) levels, hampering the normal mitochondrial activity.^[^ ^16^ ^]^ The heme group is known to exhibit peroxidase‐like activity, enabling it to catalyze the oxidation of various biomolecules in the presence of hydrogen peroxide (H_2_O_2_). This oxidative path is thought to play a crucial role in disrupting normal neurotransmission and contributing to the cognitive decline associated with the disease.^[^ ^17^ ^]^

Several factors influence the redox potential of heme, including the nature of the axial ligands bound to the iron center and the hydrophobicity of the surrounding heme environment. Several works suggest that the formation of heme‐Aβ complexes results in an increase in the peroxidase‐like activity of heme due to the axial coordination of a His residue from the peptide (most likely, via the histidine residue at position 13—His13).^[^ ^16^ ^,^ ^18^, ^19^, ^20^ ^–^ ^21^ ^]^

This mode of coordination mirrors that observed in various peroxidases, where histidine commonly serves as a proximal donor ligand to facilitate enzymatic heterolytic cleavage of O─O bond in H_2_O_2_.

**Figure 1:**
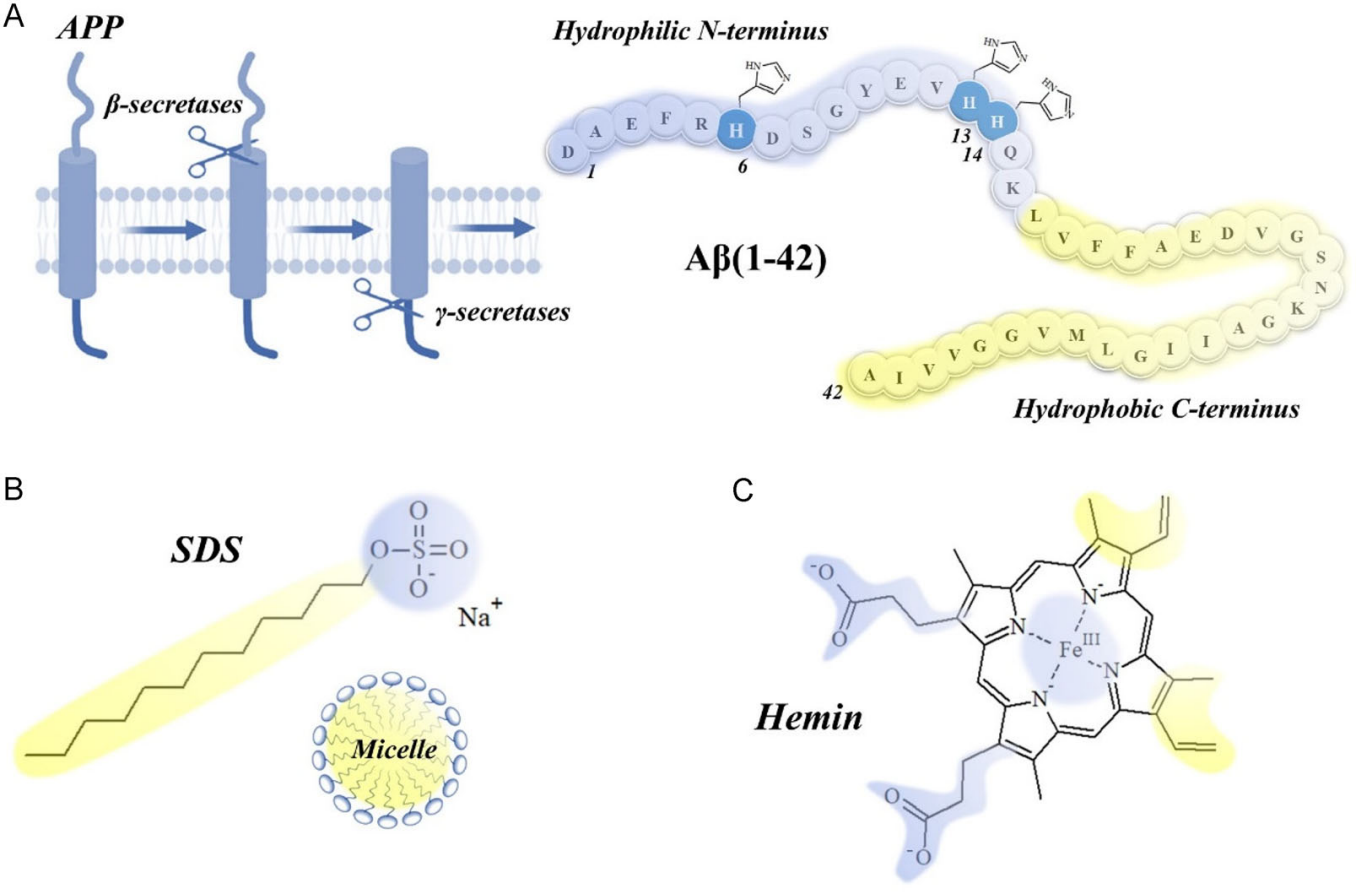
A) Cleavage of amyloid precursor protein leading to the production of amyloidogenic Aβ peptides, with a schematic representation of their sequence, which highlights three histidine residues located at the N‐terminus (which are potentially involved in hemin binding). B) Structures of SDS monomers and their organization into micelles. C) Structure of the hemin group. In all three panels, yellow and light blue highlight hydrophobic and hydrophilic/polar regions, respectively.

On the other hand, to achieve an efficient peroxidase active system, it is essential not only to have an optimal coordination sphere around the heme group, but also the presence of a hydrophobic environment can significantly modulate the activity. It is known that Aβ species are low molecular weight peptides (with a common extension of 40/42 amino acids) derived from an aberrant proteolytic cleavage of a larger transmembrane precursor protein (APP).^[^ ^22^ ^]^ This processing results in the generation of amphipathic peptides, characterized by an N*‐*terminal region prone to interact with metal ions and a more hydrophilic C‐terminal portion, which can either remain confined within the membrane or participate in intermolecular stacking, contributing to aggregation in the extracellular medium (**Figure** **1**A). Similarly, the heme group is also known to interact with cellular membranes, possibly inducing toxicity by causing changes in membrane fluidity and permeability.^[^ ^23^ ^,^ ^24^ ^]^

Therefore, it becomes crucial to better characterize the interaction between heme and membrane‐confined Aβ and, to achieve this, we have chosen to use one of the most common membrane‐like systems commonly used in such studies—micelles of sodium dodecyl sulfate (SDS).^[^ ^25^, ^26^, ^27^, ^28^ ^–^ ^29^ ^]^

The amphiphilic nature of surfactants allows to mimic lipid bilayers, which essentially consist of a hydrophobic center and a hydrophilic or polar surface. Indeed, above a specific threshold concentration (known as the critical micelle concentration (CMC), which is ≈8 mM for SDS^[^ ^30^ ^]^), the physicochemical properties of the surfactant drastically change, resulting in its self‐assembly to give spherical aggregates known as micelles. These micelles consist of a hydrophobic core formed by the hydrocarbon tails of the surfactant, surrounded by a hydrophilic (often ionic) headgroup shell, similarly to the structure of biological membranes (Figure 1B).

This environment is well‐suited to the asymmetric amphiphilic nature of the heme group, which can spontaneously interact with micelles (just as it does with membrane bilayers). Indeed, the two vinyl groups at positions 3 and 8, along with the methyl groups at positions 2 and 7 on the porphyrin ring, confer hydrophobic properties to one end of the hemin molecule, while the two polar propionate groups at positions 13 and 17 together with iron center contribute to the hydrophilic nature of the opposite end (Figure 1C).

On the other hand, there is still no general agreement regarding the precise localization of hemin within micelles.^[^ ^31^ ^]^ It seems that hemin association with the micelles is not only driven by unspecific hydrophobic interactions with the interior core but is also influenced by some polar interactions with the surfactant headgroups. Several studies have suggested that in the presence of surfactants with negatively charged headgroups (such as SDS, or sodium dodecylbenzenesulfonate), hemin predominantly exists in its monomeric form.^[^ ^32^, ^33^, ^34^ ^–^ ^35^ ^]^ The stabilization of hemin monomers in solution is likely due to the insertion of the porphyrin ring into the micelle,^[^ ^36^ ^]^ positioning it within or near the hydrophobic core (which is also consistent with the reduced size of a micelle, typically a few nanometers in diameter). In contrast, when positively charged surfactants like dodecyltrimethylammonium bromide^[^ ^34^ ^]^ are used, μ‐oxo dimers are the main species detected in solution. This indicates a different localization of the hemin group in these environments, likely more superficial (and, consequently, more accessible to give dimeric species) in the case of cationic micelles.

It is also important to note that only monomeric hemin exhibits peroxidase‐like catalytic activity, whereas aggregated species are almost entirely inactive.^[^ ^37^ ^,^ ^38^ ^]^ Consequently, the presence of a local environment that favors and stabilizes the monomeric form of hemin can significantly influence the catalysis. The presence of SDS has shown to have positive effects on the oxidative reactions toward different substrates (although depending on their chemical properties, particularly size, charge, and redox potential), increasing both the initial reaction rates and the overall reaction efficiency;^[^ ^39^ ^]^ this effect has been observed even at surfactant concentrations below the CMC and was further emphasized when the mono‐adduct hemin–histidine was used as the catalyst.^[^ ^40^ ^]^

The higher availability of catalytically active hemin monomers in membrane‐mimicking systems suggests that the interaction between hemin and biological bilayers may significantly impact neuronal damage; this occurs not only through alterations in membrane permeability but also via oxidative targeting of essential biomolecules, such as catalyzing protein modifications and degradation, lipid peroxidation, or oxidation of genetic material.

A previous work from Ghosh Dey et al. reports an analysis of the coordination and structural characterization of the heme–Aβ interaction in the presence of SDS by UV–vis, circular dichroism (CD), resonance Raman, and electron paramagnetic resonance (EPR) spectroscopies.^[^ ^29^ ^]^ This study shows that the Aβ (1–40) peptide interacts strongly with SDS micelles, but the binding of heme to Aβ is also maintained in the micellar environment by histidine coordination.

Herein, we aim to provide a detailed characterization of the heme–Aβ interaction in SDS environment by quantifying the affinity constants of the resulting complexes (both in the presence of pseudomicelles and fully formed micelles) using a combination of spectroscopic techniques, including CD and UV–vis absorption. We also aim at evaluating the peroxidase‐like activity of the complex that has been tested by previous work by Ghosh Dey et al., but without a quantitative analysis.^[^ ^29^ ^]^ In particular, we have focused our investigation on the Aβ (1–16) fragment since this N‐terminal region is known to be directly involved in metal interaction.^[^ ^41^ ^]^ Moreover, we excluded the C‐terminal tail (17–40), based on the assumption that its inclusion might simply contribute to further stabilization of the system within the membrane, rather than providing distinct mechanistic insight.

## 2 Results and Discussion

### 2.1 UV–Vis and CD Studies on Hemin Interaction with Aβ Species

When hemin is dissolved in aqueous buffered solution at physiological pH, its Soret band (or *γ* band) is characterized by an asymmetric band with maximum at 390 nm with an intensity that is strictly influenced by the degree of aggregation; indeed, in protic solvents, hemin gives rise to a mixture in equilibrium of monomers and *π*–*π* dimers and the ratio of these two species depends on multiple factors, i.e. pH, ionic strength, hemin concentration, temperature, and the presence of additives able to change the solvent properties (for example, micelles).^[^ ^42^, ^43^ ^–^ ^44^ ^]^ In this work, we intend to investigate how the presence of SDS affects the interaction between hemin and Aβ (1–16) that has been elucidated in our previous analysis by UV–vis spectrophotometric titration.^[^ ^45^, ^46^ ^–^ ^47^ ^]^

All titrations were performed by diluting the hemin stock solution (prepared as indicated in the Supporting Information) to phosphate buffer (50 mM, pH 7.4, room temperature) and, once reached the stabilization of the Soret band (generally obtained after 30 min under stirring), the peptide was added and the respective spectrum was registered after 30 s.

As observed in previous works, imidazole groups of histidine‐containing peptides are strong coordinating groups able to easily replace water or hydroxide or chloride ions in the axial positions of iron(III) porphyrin centers; in terms of optical properties, their coordination initially leads to a decrease in the contribute at 390 nm, which corresponds to the gradual formation of a penta‐coordinated high‐spin species and this reaction is governed by a binding constant indicated as *K*_1_; then, at higher concentration of ligand, the equilibrium is shifted toward the generation of a six‐coordinated low–spin complex which absorbs approximately at 410 nm and is ruled by the *K*_2_ constant.

**Figure 2:**
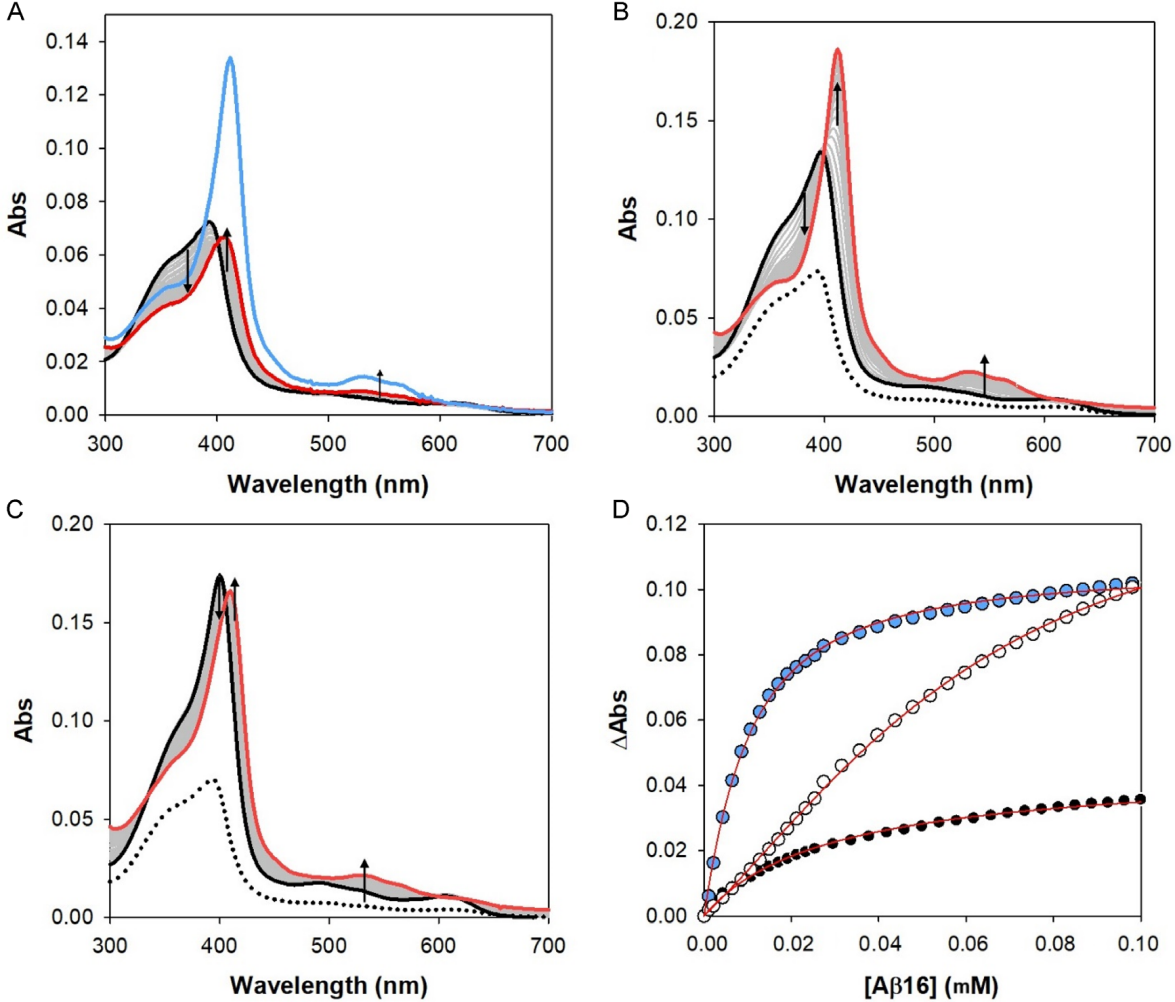
A) UV–vis spectrophotometric titration of hemin (2 µM black spectrum, after stabilization) in 50 mM phosphate buffer at pH 7.4, against increasing equiv. of A*β* (1–16) (0–50 eqv.); red spectrum is the final point of the titration, while the final addition of SDS (10 mM) is shown in light blue. Panels B**,**C) show similar titrations of hemin versus A*β* (1–16) performed after the addition of SDS (B—2 mM, and C—10 mM) at the beginning of the analysis; the dotted black spectrum represents the stabilized Soret band in only the buffered solution. D) Absorbance changes with respect to free hemin at 424 nm with subtraction of the contribution at 390 nm (for points shown in blue and black) or at 418 nm with subtraction of the contribution at 396 nm (for the data points shown in white) versus A*β* (1–16) concentration fitted by the two steps low affinity equation described in the Supporting information. Experimental points are shown as black circles for A*β* (1–16) binding in buffered solution, light blue circles for the titration which contains 2 mM SDS and white circles for that in 10 mM SDS. Red lines correspond to the calculated fittings.

The titration shown in **Figure** **2**, panel A, confirms this trend, suggesting the presence of the low‐spin species when the peptide is in large excess versus hemin (50 equiv.). To understand the effect of micelle on the hemin/Aβ interaction, SDS was added to the solution at micellar concentration (10 mM) and it results in a significant enhancement of the absorption of this species, without relevant band shifts (see light blue spectrum in Figure 2A), indicating a higher tendency for low–spin complex formation.

When the titration has been performed starting with SDS in submicellar amounts (2 mM ‐ Figure 2, panel B), the band of the not peptide‐bound hemin shifts to 400 nm, a typical value observed with monomeric hemin (as with hemin dissolved in aprotic solvents like dimethyl sulfoxide (DMSO)^[^ ^48^ ^]^). This effect is evident not only when the micelles are fully formed, but also occurs in the presence of millimolar, submicellar concentrations (Figure S1, Supporting Information). As stated before, the high solubility of porphyrins in the presence of SDS is primarily related to hydrophobic interactions between the porphyrin ring and the lipophilic tails of the surfactant (as formed micelles or incomplete pseudomicelles), which can compete with the porphyrin–porphyrin attractive forces and contribute to keep hemin in a nonaggregated state.^[^ ^31^ ^,^ ^36^ ^]^ This alteration in the aggregation state of hemin in solution enhances its accessibility to peptide interactions, thus promoting the formation of penta‐ and hexa‐coordinated hemin–Aβ species, as indicated by higher *K*_1_ and *K*_2_ values in **Table** **1**. However, when SDS reaches its CMC, the hemin group is primarily confined within the micelles, making it harder for the Aβ (1–16) peptide (which is mainly hydrophilic) to access the iron center.

**Table 1:** Equilibrium constants for hemin interaction to Aβ (1–16) peptide in 50 mM phosphate buffer at pH 7.4 at 25 °C, also in the presence of SDS (2 and 10 mM, submicellar and micellar concentrations, respectively).

Peptide	*K*_1_ [M^−1^]	Log*K*_1_	*K*_2_ [M^−1^]	Log*K*_**2**_
Aβ (1–16)	(6.41 ± 0.23) × 10^4^	4.81	(1.27 ± 0.05) × 10^4^	4.10
+2 mM SDS	(1.15 ± 0.05) × 10^5^	5.06	(4.36 ± 0.65) × 10^4^	4.64
+10 mM SDS	(7.74 ± 0.50) × 10^3^	3.88	(2.17 ± 0.23) × 10^4^	4.34

This significantly restricts the first peptide molecule's access to the iron center, resulting in an important decrease in *K*_1_ values, while *K*_2_ exhibits a value intermediate between that of hemin binding in buffered solution alone (where hemin is partially in its dimeric form) and that in the presence of 2 mM SDS (fully monomeric and highly accessible) (Table 1).

We also compared the interaction of hemin with ligands that have lower steric hindrance, such as imidazole (Im), in a buffered solution versus in the presence of SDS. Although 100 equivalents of imidazole are not sufficient to fully saturate hemin binding in aqueous solution (as shown in Figure S2, panel A, Supporting Information), since it is a weaker ligand for hemin with respect of the imidazole group of a His residue in peptides, we observed that the presence of SDS appears to influence the hemin/Im interaction by reducing the affinity (Figure S2, panel D, Supporting Information). This can be explained by considering that although the high concentration of hemin monomers in solution promoted by the surfactant would favor Im binding, Im may interact more strongly with SDS compared to the Aβ peptide, resulting in partial sequestration of the ligand.

Anyway, in the presence of a large excess of Im (1000 eqv.), its concentration within the micelle rises, by promoting the coordination of hemin and the formation of low‐spin species trapped within the SDS micelles. This result partially mirrors the behavior observed with the peptide, although the two ligands differ significantly in terms of hemin binding affinity, net charge, and molecular size (Figure S3, Supporting Information).

**Figure 3:**
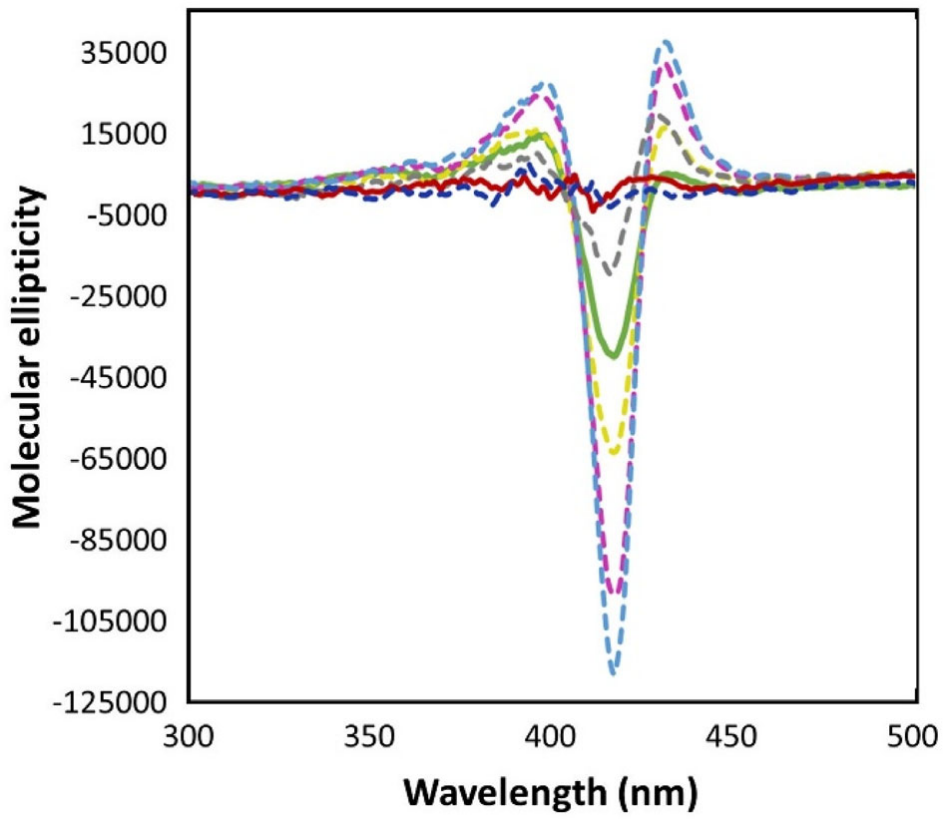
CD vis spectra of hemin solution (30 µM—red spectrum) in 5 mM phosphate buffer at pH 7.4, with the addition of A*β* (1–16) (300 µM—green) and SDS (0.5 mM, dashed yellow; 1 mM, dashed violet; 2 mM, dashed light blue; 5 mM, dashed gray; and 10 mM, dashed blue).

The interaction between hemin and Aβ (1–16) in SDS micelle is further analyzed by the CD visible spectra shown in **Figure** **3**. The hemin–Aβ (1–16) complex exhibits typical spectral features when low‐spin species are present in solution, whereas high‐spin species or ‘uncoordinated’ hemin are CD silent.^[^ ^49^ ^]^ When SDS is present at submicellar concentrations (up to about 2 mM), the CD signal intensity of the hemin‐ (Aβ (1–16))_2_ complex increases, reflecting the greater concentration of monomeric heme in solution, which is accessible for coordination with the peptide (as also suggested by the *K*_1_ and *K*_2_ values which rules the complex formation). In contrast, when SDS forms micellar structures, the CD signal diminishes. This effect is probably not due to the dissociation of the Aβ (1–16) peptide (vide infra) but rather may suggest a sequestration of the hemin complex within the micelle. However, it is likely that the peptide does not fully penetrate the micelle, instead remaining partially exposed on the outside. That would mean that the peptide‐bound hemin group, buried within the hydrophobic region of the micelles and free to rotate, cannot feel the chiral environment given by the peptide.

Similar results are observed when the peptide is added after hemin has already been trapped by the micelles (Figure S4, panel B), where the micellar SDS completely abolishes the characteristic CD signal of the six‐coordinated low–spin complex, while the UV–vis absorption spectrum with absorption maximum around 414 nm of this form is retained. On the other hand, far UV CD spectra of the peptide solution, with and without SDS (10 mM), provide evidence that the peptide is not fully interacting with the micelle, and likely, the interaction with SDS is limited to the residues neighboring the coordinating His. In fact, only a slight structural change toward an alpha‐helix in the Aβ peptide occurs after the addition of 10 mM SDS, while submicellar concentrations of SDS do not alter the random structure of the peptide (Figure S4, panel A, Supporting Information). Furthermore, the addition of hemin, and thus the formation of the complex, does not induce any significant structural effect. Similar results were obtained by Ghosh et al. for the interaction by CD spectroscopy of hemin with Aβ (1–40) in the far‐UV region;^[^ ^29^ ^]^ this similarity between the truncated peptide Aβ (1–16) and the Aβ (1–40) isoform reinforces the initial hypothesis that the two peptides, despite having an interaction with a different strength with the micelle, lead to the same behavior.

Therefore, once micelle formation is complete, hemin becomes encapsulated in the micelle, maintaining the coordination with the peptide, which interacts with the polar groups of the micelles. Under these conditions, the wrapping of the peptide chain around the porphyrin is prevented, the chirality of the metal center is annulled, and the dichroic signal of the complex is completely turned off. The not‐bound peptide, however, is mainly located outside the lipid structure, likely adhering to the surface.

### 2.2 Peroxidase‐Like Activity Studies of Hemin/Aβ Species

The ability of SDS to keep hemin in its monomeric state, to hide it within the micelles, and to modulate its axial coordination, based on the accessibility of the metal center to ligands, can directly affect the hemin peroxidase‐like activity.

To assess the effect of SDS on the catalytic potential of hemin/Aβ complexes, we compared the previously reported^[^ ^47^ ^]^ pseudoperoxidase activity of hemin–Aβ (1–16) complex in phosphate buffer at pH 7.4 toward two standard substrates, the phenol 3‐(4‐hydroxyphenyl)propanoic acid (HPA) and the catechol dopamine (DA) with that in the presence of different SDS concentrations.

We first evaluated how the reaction rate for both substrates is affected by peptide concentration, with the goal of optimizing the formation of the 1:1 hemin/peptide five‐coordinated species. This ensures that one coordination position on the iron remains free to react with hydrogen peroxide, thereby achieving the maximal catalytic activity of the system.

**Figure 4:**
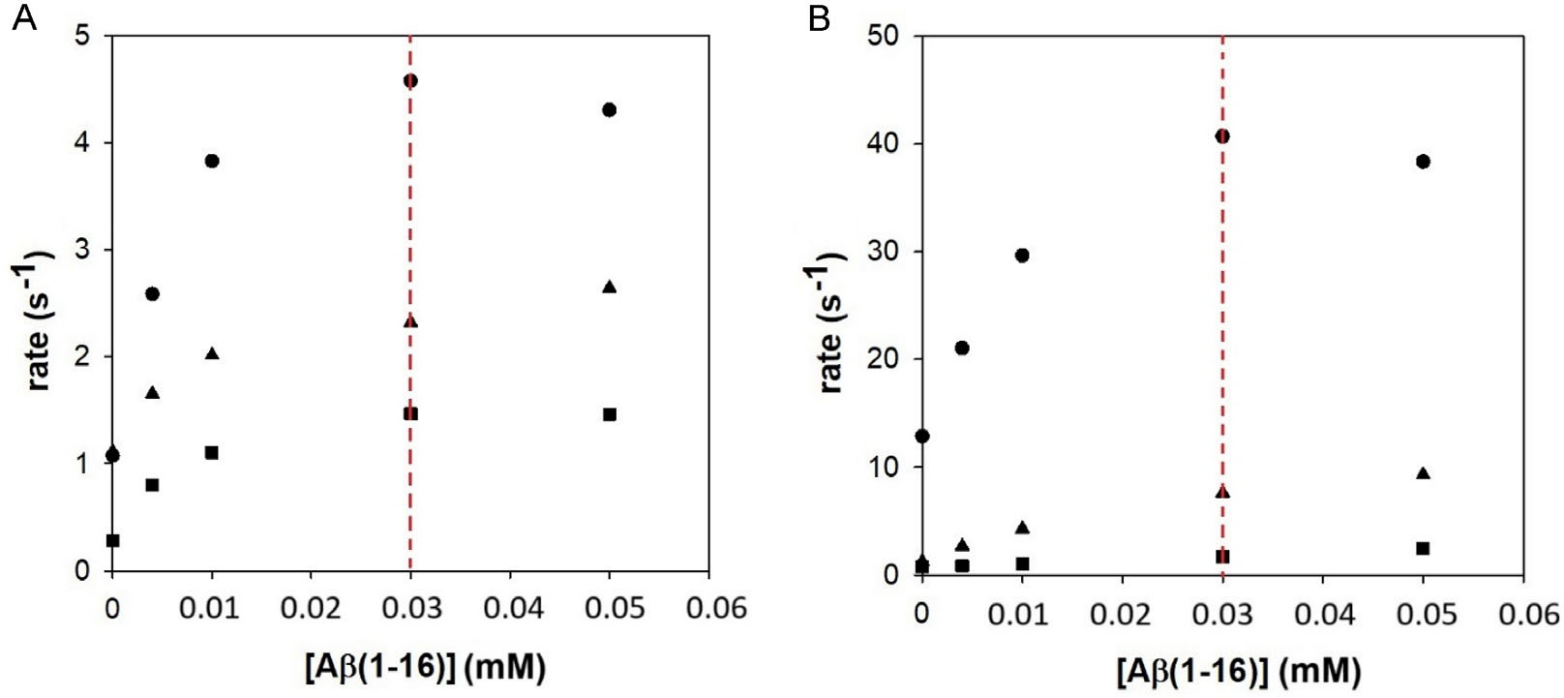
Plots of the initial oxidation rates of HPA (A—1 mM) and DA (B—1 mM) oxidation versus [A*β* (1–16)] (0–50 μM) in the presence of hydrogen peroxide (A—40 mM and B—100 mM) and hemin (A—2 μM and B—0.2 μM), in 20 mM phosphate buffer solution at pH 7.4 (circles), with the addition of SDS (2 mM—triangles and 10 mM—squares).

As already observed in the previous analysis,^[^ ^47^ ^]^ the presence of the Aβ peptide results in an increase in hemin activity; however, at concentrations above 30 µM, the system reaches a maximum in activity, after that the activation of hydrogen peroxide becomes less efficient (**Figure** **4**). The “bell shape” profile becomes less apparent when SDS is introduced into the reaction mixture, both at submicellar and micellar concentrations. This is probably due to the lower polarity around the hemin induced by SDS that reduces the acid/base effect required for the activation of hydrogen peroxide; in fact, as shown below, the presence of SDS significantly quenches the peroxidase‐like reactivity. SDS gives rise, as a further effect, to an increased formation of low‐spin species (higher K_2_ values) that are catalytically inactive; however, this could be seen as a minor effect since it would imply a higher peroxide concentration to reach saturation.

On the other hand, the extent of this effect varies depending on the chemical properties (particularly, the charge) of the substrate. When HPA (negatively charged, Figure S5, Supporting Information) or DA (positively charged, Figure S6, Supporting Information) is used as substrates, a different kinetic trace pattern of oxidation is observed. In fact, independently of the peptide coordination, the catalytic efficiency also accounts for the different interactions of the substrate with the SDS environment (in which hemin is trapped). The surfactant appears to negatively affect the first step of the kinetic profiles (with both substrates, but particularly with the positively charged DA molecule, probably due to a strong interaction with the sulfate group), while it seems to stabilize the accumulation of some oxidative products (likely more hydrophobic than reagents) as indicated by the higher slopes in the second phase compared to the ones obtained with hemin in buffer alone (Figure S7, Supporting Information).

Once characterized, the best hemin: Aβ ratio, we evaluated the reactivity efficiency of the heme complexes toward hydrogen peroxide in terms of formation of the high‐valent iron species, as shown in Equation (1).

(1) $$\mathrm{HM}+H_{2}O_{2}⇄\left[\mathrm{HM}/H_{2}O_{2}\right]\;{\text{→ HM}}_{\mathrm{ox}1}+H_{2}O$$

(2) $${\mathrm{HM}}_{\mathrm{ox}1}+{\text{SH→HM}}_{\mathrm{ox}2}+S^{•}+H^{+}$$

(3) $${\mathrm{HM}}_{\mathrm{ox}2}+\mathrm{SH}+H^{\text{+}}\text{→ HM}+S^{•}+H_{2}O$$

where HM is the ferric heme complex, SH is a generic substrate, and HM_ox1_ and HM_ox2_ are high‐valent iron species, analogous to peroxidase Compound I and Compound II.

Recent studies by Ghosh Dey et al*.* confirm that the hemin–Aβ peptide complex behaves in the same way, thanks to the characterization of the formation of Compound I and Compound II,^[^ ^50^ ^]^ and the iron(III)‐hydroperoxo adduct that tends to undergo the cleavage of the O─O bond (commonly indicated as Compound 0).^[^ ^51^ ^]^

**Figure 5:**
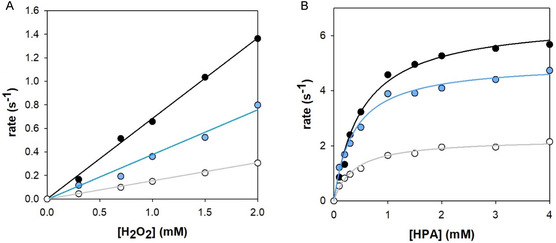
Initial oxidation rates of HPA in 20 mM phosphate buffer solution at pH 7.4 and 25 °C in the presence of A) variable amounts of hydrogen peroxide (0–2 mM) and fixed [substrate] (1 mM), or B) fixed [H_2_O_2_] (40 mM) and variable amounts of substrate (0–4 mM), with the addition of hemin (2 μM), A*β* (1–16) (30 µM, black circles) and SDS (2 mM—light blue, and 10 mM—white).

Indeed, by studying the peroxidase‐like reaction with a fixed substrate concentration in moderate excess over the hemin complex, the reaction rates are linearly dependent on the concentration of hydrogen peroxide. Equation (1) becomes the rate‐determining step, characterized by the rate constant k_1obs_, while the following substrate oxidation steps occur rapidly. The rate constants k1_obs_ for the hemin complexes were calculated from the slopes, shown in **Figure** **5**, panel A, of kinetic traces (Figure S8, Supporting Information) versus [H_2_O_2_], with data provided in **Table** **2**.

**Table 2:** Kinetic constants for the HPA oxidation promoted by hemin–Aβ complexes in the presence of H_2_O_2_ in 20 mM phosphate buffer at pH 7.4 and 25 °C.

		*k*_1obs_ [M^−1^ s^−1^]	*K*_M_ [mM]	*k*_cat_ [s^−1^]	*k*_cat_/*K*_M_ [M^−1^s^−1^]
Hemin alone^a)^		103 ± 4	0.80 ± 0.09	1.29 ± 0.05	(1.58 ± 0.02) × 10^3^
+A*β* (1–16)	in only buffer^a)^	439 ± 8	0.43 ± 0.03	7.48 ± 0.14	(1.73 ± 0.02) × 10^4^
	with 2 mM SDS	353 ± 31	0.40 ± 0.04	5.06 ± 0.13	(1.26 ± 0.18) × 10^4^
	with 10 mM SDS	136 ± 2	0.31 ± 0.05	2.05 ± 0.07	(6.61 ± 0.11) × 10^3^

^a)^ The kinetic parameters of hemin alone and hemin–Aβ (1–16) were taken from Bacchella et al.^[^ ^47^ ^]^

Hydrogen peroxide activation appears to be more efficient in the absence of SDS, and particularly, when SDS reaches its critical micellar concentration, the *k*_1obs_ values are significantly reduced. However, it is unlikely that this effect is due to the inaccessibility of hydrogen peroxide to the complex trapped in the membrane, as hydrogen peroxide is a diffusible species capable of penetrating the micelle. This is also confirmed by the similar sensitivity of hemin to degradation catalyzed by hydrogen peroxide, whether hemin is free in solution or trapped in a micelle (Figure S9, Supporting Information).

We believe that the whole effect is given by two principal reasons: 1) SDS has a negative effect in the rate of the acid/base proton transfer required for the transformation of the iron‐bound peroxide into the high‐valent iron‐oxo species of (Equation 1); and 2) the increased formation of the low‐spin species compared to catalytically active penta‐coordinated species, negatively affects the peroxidase‐like efficiency of the system.

Then, in order to analyze the catalytic reaction steps involved in the substrate oxidation, it is essential that the reaction between hemin and hydrogen peroxide occurs faster than the reduction of the high‐valent iron species by the substrate. This requires operating under saturating hydrogen peroxide conditions (Figure S10, Supporting Information): under such conditions, the substrate oxidation becomes the rate‐limiting reaction for the turnover process (described in (Equation 3), since HM_ox1_ is a faster oxidant than HM_ox2_), thus allowing the determination of *k*_cat_ and *K*_M_ values.

Although the *K*_M_ values of the hemin/Aβ complex suggest a slight promotion, depending on the presence of SDS, of the interaction between the catalyst and the substrate, the turnover values (*k*_cat_/*K*_M_) presented in Table 2 indicate an important reduction in the catalytic efficiency of the complex, particularly evident when SDS is micellar (where the catalytic efficiency is halved). The minor reactivity in the presence of SDS can be also deducted by the comparison of the absorption spectra of the oxidized product taken during the kinetic spectra (Figure S11, Supporting Information).

The catalytic studies shown above and performed with the substrate HPA were also repeated with another oxidizable substrate, dopamine (DA), which has been extensively used in our previous research.^[^ ^47^ ^,^ ^52^ ^,^ ^53^ ^]^

**Figure 6:**
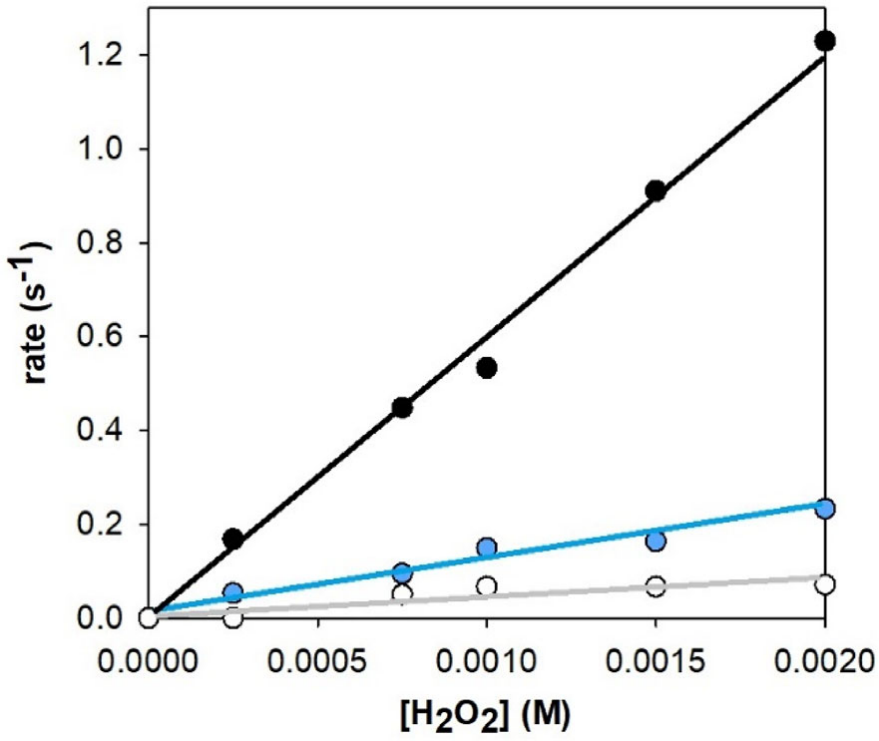
Initial oxidation rates of DA in 20 mM phosphate buffer solution at pH 7.4 and 25 °C in the presence of variable amounts of hydrogen peroxide (0–2 mM) and fixed [substrate] (1 mM), with the addition of hemin (0.2 μM), A*β* (1–16) (30 µM, black circles), and SDS (2 mM—light blue, and 10 mM—white).

The rate constants *k*_1obs_ for the hemin complexes were determined from the slopes shown in **Figure** **6**, which are obtained from kinetic profiles at different hydrogen peroxide concentrations and at fixed [substrate] and [catalyst] (Figure S12, Supporting Information). **Table** **3** shows a significant reduction in hydrogen peroxide activation when the surfactant is present in the reaction mixture, regardless of whether SDS is in its micellar form or not.

**Table 3:** Kinetic constants for the DA oxidation promoted by hemin–Aβ complexes in the presence of H_2_O_2_ in 20 mM phosphate buffer at pH 7.4 and 25 °C.

		*k*_1obs_ [M^−1^ s^−1^]
Hemin alone^a)^		247 ± 11
+A*β* (1–16)	in only buffer^a)^	622 ± 32
	with 2 mM SDS	109 ± 10
	with 10 mM SDS	40 ± 10

^a)^ The kinetic parameters of hemin alone and hemin–A*β* (1–16) were taken from Bacchella et al.^[^ ^47^ ^]^

As mentioned above, the reactivity efficiency of the system is influenced not only by the interaction between the catalyst and the surfactant, but it is also strongly modulated by the interactions (mainly, electrostatic) between the negatively charged sulfate heads of SDS and the charged groups of the substrate.^[^ ^39^ ^]^ With DA, these interactions hinder the intramolecular cyclization to dopaminochrome, which is the product spectrophotometrically visualized during catalysis.^[^ ^54^ ^]^

The strong interaction between DA and SDS significantly modulates the already complex reaction mechanism, as evidenced in our recent study.^[^ ^47^ ^,^ ^55^ ^]^ Indeed, at high concentrations of DA, the catalytic efficiency of both hemin and hemin–Aβ complexes is partially inhibited. However, this substrate inhibition effect is not complete, as the oxidation rate does not drop to zero (Figure S13 and S14, Supporting Information). This behavior diverges from the typical Michaelis–Menten trend observed with other oxidizable substrates, such as HPA, as shown earlier,^[^ ^47^ ^]^ and this can be explained by assuming that under saturating conditions, two molecules of substrate bind to the catalyst, and, in this form, the reactivity is significantly reduced.

Unfortunately, this peculiar reaction mechanism complicates the kinetic studies and the determination of reliable *k*_cat_ and *K*_M_ values when SDS is present in the reaction medium.

## 3 Conclusion

The interaction between hemin, Aβ peptide, and cell membranes presents a complex interplay that is crucial for understanding the etiology of neurodegenerative conditions, particularly AD. Hemin dyshomeostasis is implicated in exacerbating the pathological effects of neurodegenerative diseases through various mechanisms, including oxidative stress^[^ ^56^ ^]^ and membrane destabilization.^[^ ^57^ ^,^ ^58^ ^,^ ^59^, ^60^ ^]^ In the present study, we report the characterization of how the presence of SDS micelles affects the binding and the reactivity of the complex between hemin and Aβ (1–16) peptide. This membrane‐like environment has been chosen because the binary interactions of hemin‐SDS and Aβ‐SDS have been widely characterized. Our analysis confirms that the peptide is unable to deeply penetrate the micelle, whereas hemin can, but this effect is largely dependent on the SDS micelle concentration. More in detail, submicellar SDS concentrations favor the presence of hemin in the monomeric form in aqueous solution, making it more available to interact with the peptide. This effect is evident in both the formation of 1:1 hemin (high‐spin iron) : Aβ complex and 1:2 hemin (low‐spin iron): Aβ complex (as indicated by the increased *K*_1_ and *K*_2_ values compared to the situation without SDS). When the SDS concentration is higher than CMC, monomeric hemin tends to be encapsulated inside the core, and the binding with the first Aβ (1–16) peptide becomes more difficult (low *K*_1_ value). However, the binding of a second peptide is still possible, as shown by the typical low‐spin iron absorption band at 414 nm and a relatively high *K*_2_ value. However, the hexa‐coordinated hemin iron complex in the presence of the micelle does not show optical features in the visible region of the CD spectra (contrary to the same complex in aqueous solution), indicating that a different conformation of the peptide makes the binding with hemin more flexible. The evidence that the hemin: Aβ complex is not totally trapped in the micelle has also been proposed by Ghosh Dey et al.^[^ ^29^ ^]^ that report that the formation of low‐spin hexa‐coordinated species can be perturbed by the addition of copper (II) that induces a transition to the high‐spin five‐coordinated iron complex, although in that case, the study was conducted with the Aβ (1–40) isoform, which guarantees a stronger interaction with the micelle having the hydrophobic tail.

Moreover, the presence of SDS (in both submicellar and CMC concentrations) diminishes the peroxidase‐like reactivity of hemin–Aβ (1–16) complex. For the HPA oxidation analysis, this effect is remarkable for both *k*_1obs_ (which rules the rate of hydrogen peroxide activation) and *k*_cat_ (which is referred to assubstrate oxidation) rate constants. Also, in the presence of the catecholamine DA as substrate, the reactivity of hemin–Aβ (1–16) complex is lowered in the presence of SDS. Remarkably, this decrease is observed for both the positively charged (DA) and the negatively charged (HPA) substrate.

In conclusion, this study suggests that a membrane‐like environment plays a critical role in modulating the interplay between Aβ and hemin, affecting both complex formation and the oxidative reactivity of the resulting complex. Our data indicate that this interaction partially inhibits the formation of the hemin–Aβ complex and, more importantly, it limits its oxidative capacity. This effect is considerably by using the short Aβ (1–16) and we expect that it would be potentiated in the presence of the full‐length 1–40 peptide. Indeed, the inclusion of the C*‐*terminal tail (17–40) might simply contribute to further stabilize the complex within the membrane, without providing distinct mechanistic insight from the short Aβ (1–16) fragment. The present analysis represents a further step in the comprehension of the cross‐talk between these components, which is vital for understanding the mechanisms underlying neurotoxicity in AD.

## Conflict of Interest

The authors declare no conflict of interest.

## Supporting information

Supplementary Material

## Data Availability

The data that support the findings of this study are available from the corresponding author upon reasonable request.
