# Supplementary material for: How Sodium Dodecyl Sulfate Micelles Affect the Coordination and Peroxidase‐Like Activity of the Hemin–Aβ16 Complex
Source: Chempluschem. 2025 Aug 6;90(10):e202500304. doi: 10.1002/cplu.202500304 (PMC12509477; doi:10.1002/cplu.202500304)
Supplement: Supplementary file 1 — Supplementary Material [file CPLU-90-e202500304-s001.pdf]

Supporting information

How sodium dodecyl sulfate (SDS) micelles affect the coordination and peroxidase-like activity of the hemin-Aβ16 complex

Chiara Bacchella,*[a] Simone Novellini,[a] Elisa Miotto,[a] Stefania Nicolis,[a] Enrico Monzani,[a] and Simone Dell'Acqua*[a]

[a] Dr. C. Bacchella, S. Novellini, E. Miotto, Dr. S. Nicolis, Prof. E. Monzani, Prof. S. Dell'Acqua
Dipartimento di Chimica
Università di Pavia
Via Taramelli 12, 27100 Pavia, Italy
E-mail: chiara.bacchella@unipv.it ; simone.dellacqua@unipv.it

General Procedures.

Fmoc-amino acids, Rink amide resin, and other reagents for SPPS were purchased from Novabiochem. Other chemicals, unless specified, were reagent grade from Merck, with hemin obtained from Alfa Aesar. Semi-preparative HPLC purification of Aβ(1-16) peptides was performed using a Shimadzu Prominence instrument, equipped with a degassing unit (DGU-20A3R), two LC-20AD pumps, and a SPD-M20A diode array detector (working range: 190–800 nm), utilizing a Phenomenex Jupiter C12 Proteo 90A column (250 × 10 mm). Mass spectra and LC-MS/MS data were obtained using an LCQ ADV MAX ion-trap mass spectrometer with an ESI ion source. The system was operated in automated LC-MS/MS mode with a Surveyor HPLC system (Thermo Finnigan, San Jose, CA, USA) equipped with a Phenomenex Jupiter Proteo column (4 µm, 150 × 2.0 mm). HPLC-MS data analysis was conducted using Bioworks 3.1 and Xcalibur 2.0.7 SP1 software (Thermo Finnigan, San Jose, CA, USA). UV-vis spectra and kinetic data were recorded on an Agilent 8453 diode array spectrophotometer with a thermostated, magnetically stirred optical cell. Circular dichroism (CD) spectra were acquired using a Jasco J-1500 spectropolarimeter with a 1 cm path length quartz cell. Hemin stock solutions were prepared by dissolving hemin in 0.1 M NaOH in Milli Q water via sonication for several minutes, followed by centrifugation at 14,000 rpm for 2 minutes. Hemin concentrations were determined by UV-vis spectroscopy using a molar extinction coefficient (ε_{390} 65000 $M^{-1}cm^{-1}$) obtained via the hemochromogen assay.[48] Aβ peptide (1–16) ($_1$DAEFRHDSGYEVHHQK$_{16}$-NH_2) was synthesized by traditional Fmoc-SPPS in DMF, as previously described. [59]

Hemin/Aβ interaction studies.

Spectrophotometric titrations were conducted with hemin (2 µM) in 50 mM phosphate buffer at pH 7.4 and Aβ peptide solutions to assess the equilibrium constants for peptide interaction. These experiments were carried out in thermostated cells at 25 °C. The titration data were analyzed using Fig.P Software (Version 2.2a; Durham, NC, USA: 1994), utilizing the absorbance values at 424 and 390 nm for the Soret band. The equilibrium constants were calculated by plotting the absorbance changes relative to free hemin (corrected for dilution) against the added ligand equivalents. Data fitting enabled the determination of equilibrium constants for the two-step, low-affinity, binding processes, (K_1) (which rules Step 1: [hemin] + Aβ ⇄ [hemin(Aβ)] and (K_2) (for Step 2: [hemin(Aβ)] + Aβ ⇄ [hemin(Aβ)$_2$].
The equation used for determining the K_1 and K_2 values for the generation of hemin:Aβ complexes (1:1 and 1:2) is shown below:

$$\Delta\text{Abs} = \frac{(A_1K_1\mathrm{x} + A_2K_1K_2x^2)}{(1{+}K_1\mathrm{x} + K_1K_2x^2)}$$

where ΔAbs, absorbance changes with respect to free hemin at 424 nm with subtraction of the contribution at 390 nm; A_1 and A_2, absorbance differences with respect to free hemin for the 1:1 and 1:2 hemin:Aβ complexes, respectively; K_1, binding constant for the formation of 1:1 hemin:Aβ complex; K_2, binding constant related to the formation of 1:2 hemin:Aβ complex from the 1:1 complex; x, peptide concentration after each addition.
Hemin binding was also studied in the presence of the smaller ligand, imidazole, that allows to reach a large excess (0-100/1000 eqv.) in the same buffered solution. Titrations of hemin with both Aβ or imidazole were also performed by first adding a fixed amount of SDS (2 and 10 mM) to the hemin solution (2 µM) in 50 mM phosphate buffer at pH 7.4, before introducing the coordinating species.

Supporting information

Characterization of hemin-Aβ complexes via Circular Dichroism (CD).

The conformational properties of the Aβ(1-16) peptide were assessed in the 192-280 nm range with a Jasco 1500 spectrometer, in a 1 cm path length cell. Aβ solution (8.5 μM) in 5 mM phosphate buffer at pH 7.4 was analyzed prior and after the addition of hemin (8 μM) and SDS (2 and 10 mM). CD spectra of the peptide alone and of the hemin-Aβ complex were recorded with five acquisitions. Visible CD spectra, spanning from 250 to 500 nm, were collected for samples containing 30 μM hemin, 300 μM Aβ(1-16) and increasing amounts of SDS (0-10 mM) in 5 mM phosphate buffer at pH 7.4.

Catalytic activity of hemin/Aβ species *vs* HPA and DA.

The kinetic studies were conducted at 25 °C in 20 mM phosphate buffer at pH 7.4. Substrate oxidation kinetics were obtained by adding hydrogen peroxide to a mixture containing HPA or DA (1 mM), hemin (2 μM), and Aβ(1-16) (0-50 μM). The oxidative reaction was monitored by following the formation of the dimeric product of HPA at 300 nm (ε_{300} = 1950 $M^{-1}cm^{-1}$) or DAQ at 475 nm (ε_{475} = 3300 $M^{-1}cm^{-1}$).[54,60]
To determine the kinetic constants of the hemin complexes, the reaction rates (obtained from the initial slopes of the kinetic curves) were converted into turnover rates (s^{-1}) by dividing the values by the catalyst concentration and the molar extinction coefficient of the oxidation products; for DA, the values were multiplied by a factor 2 to take into account one catalytic cycle. To obtain k_{1obs} values in the presence of HPA, the initial slopes were measured at low hydrogen peroxide concentrations from the kinetic profiles in the presence of HPA (1 mM), hemin (2 μM), and Aβ(1-16) (30 μM), with variable amounts of hydrogen peroxide (0-50 mM). For k_{1obs} determination with DA, hemin concentration was decreased at 0.2 μM, while the amounts of hydrogen peroxide were varied in the range (0-500 mM).
The substrate-dependence rate analyses for the kinetic studies with HPA were performed with hemin (2 μM), Aβ(1-16) (30 μM) and hydrogen peroxide (40 mM), varying substrate concentrations in the range 0-4 mM. The kinetic rates in the presence of HPA were fitted using the classical Michaelis-Menten equation.[47]

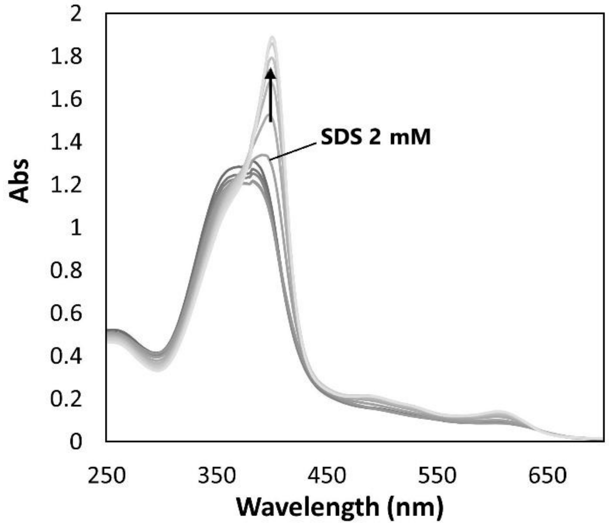

Figure S1. Absorbance changes of the Soret band of hemin solution (20 μM) in 50 mM phosphate buffer at pH 7.4 vs increasing concentrations of SDS (0-20 mM).

Supporting information

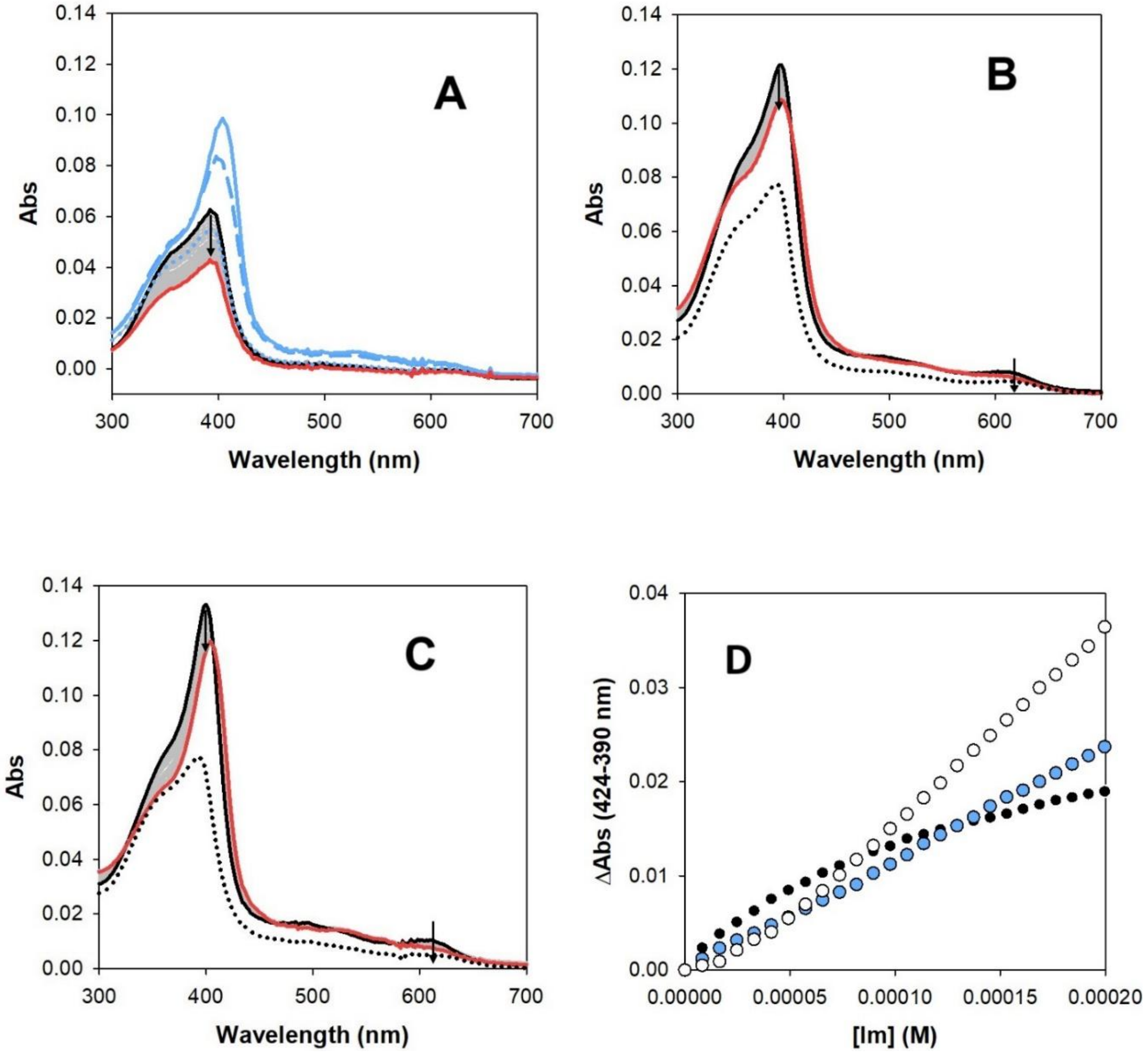

Figure S2. (A) UV–vis spectrophotometric titration of hemin (2 µM - black spectrum, after stabilization) in 50 mM phosphate buffer at pH 7.4, against increasing equiv. of imidazole (0-100 eqv.); red spectrum is the final point of the titration, while the final addition of SDS is shown in light blue (dotted, 0.5 mM; dashed, 2 mM; solid, 10 mM). Panels **B** and **C** show similar titrations of hemin *vs* imidazole performed after the addition of SDS (**B** - 2 mM and **C** - 10 mM) at the beginning of the analysis. **D)** Absorbance changes with respect to free hemin at 424 nm with subtraction of the contribution at 390 nm *vs* Im concentration; experimental points are shown as black circles for Im binding in buffered solution, light blue circles for the titration which contains 2 mM SDS and white circles for that in 20 mM SDS.

Supporting information

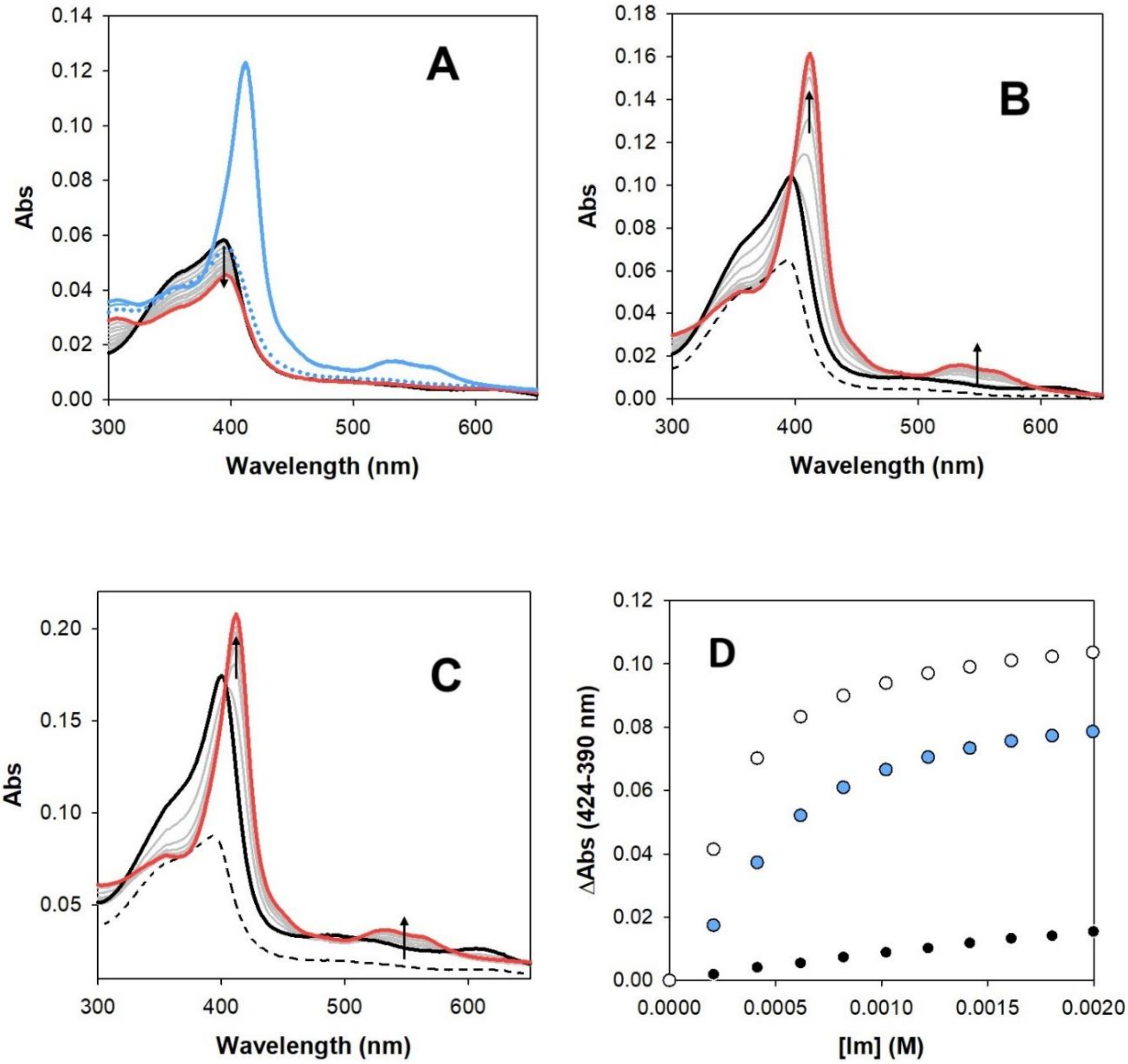

Figure S3. (**A**) UV–vis spectrophotometric titration of hemin (2 μM - black spectrum, after stabilization) in 50 mM phosphate buffer at pH 7.4, against increasing equiv. of imidazole (0-1000 eqv.); red spectrum is the final point of the titration, while the final addition of SDS is shown in light blue (dotted, 0.5 mM; solid, 10 mM). Panels **B** and **C** show similar titrations of hemin *vs* imidazole performed after the addition of SDS (**B** - 2 mM, and **C** - 10 mM) at the beginning of the analysis. **D)** Absorbance changes with respect to free hemin at 424 nm with subtraction of the contribution at 390 nm *vs* Im concentration; experimental points are shown as black circles for Im binding in buffered solution, light blue circles for the titration which contains 2 mM SDS and white circles for that in 20 mM SDS.

Supporting information

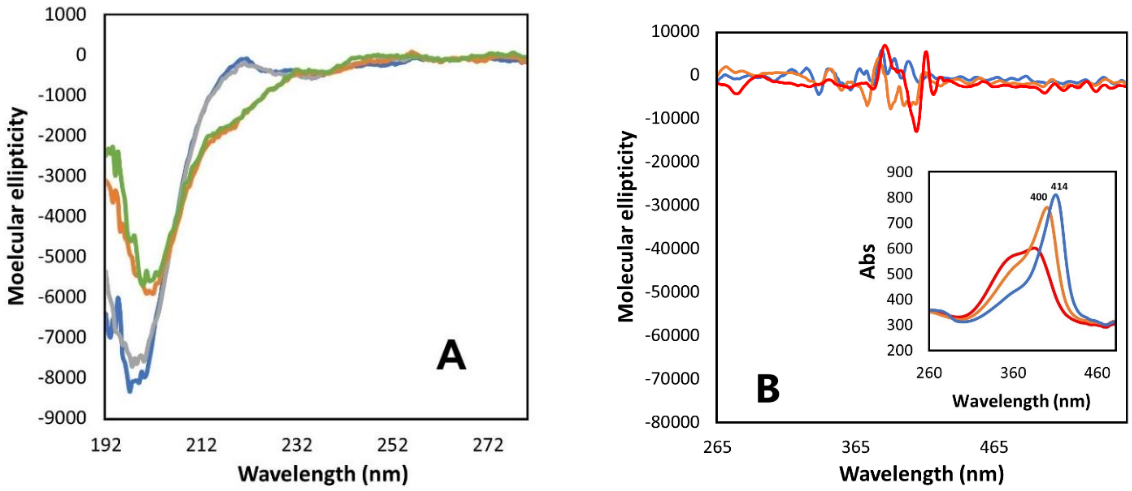

Figure S4. (A) CD far UV-spectra of Aβ16 solution (8.5 μM- blue spectrum) in 5 mM phosphate buffer at pH 7.4, with the addition of SDS (2 mM, grey and 10 mM, orange) and of hemin (8 μM- green). **(B)** CD vis spectra of hemin solution (30 μM - red) in 5 mM phosphate buffer at pH 7.4, with the addition of SDS (10 mM, orange) and Aβ(1-16) (300 μM - blue).

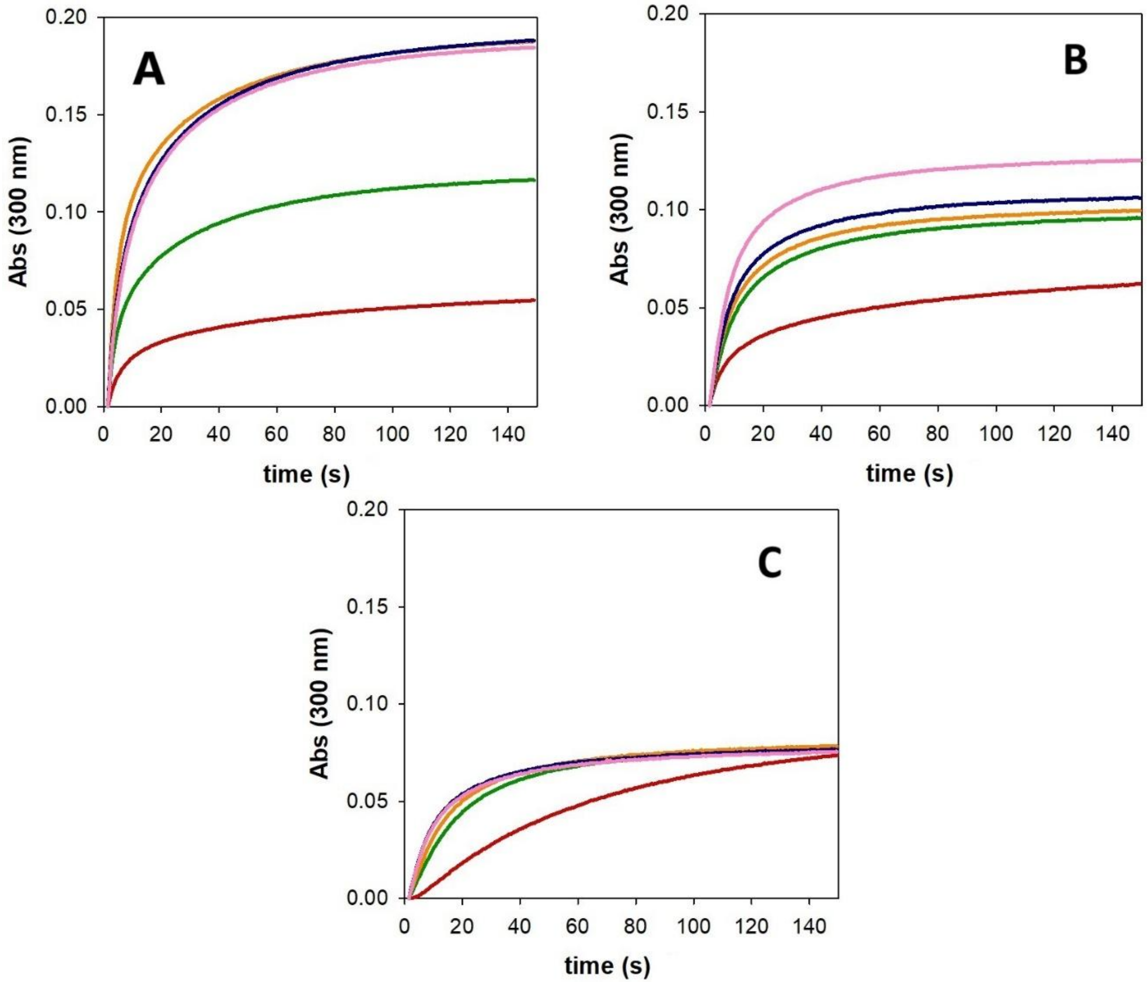

Figure S5. Kinetic profiles of HPA (1 mM) oxidation in 20 mM phosphate buffer solution at pH 7.4 and 25 °C in the presence of hydrogen peroxide (40 mM) and hemin (2 μM, red traces) with increasing equivalents of Aβ(1-16) (4 μM, green; 10 μM, orange; 30 μM, blue; 50 μM, pink); 2 mM and 10 mM SDS (panels B and C, respectively) was added to the reaction media.

Supporting information

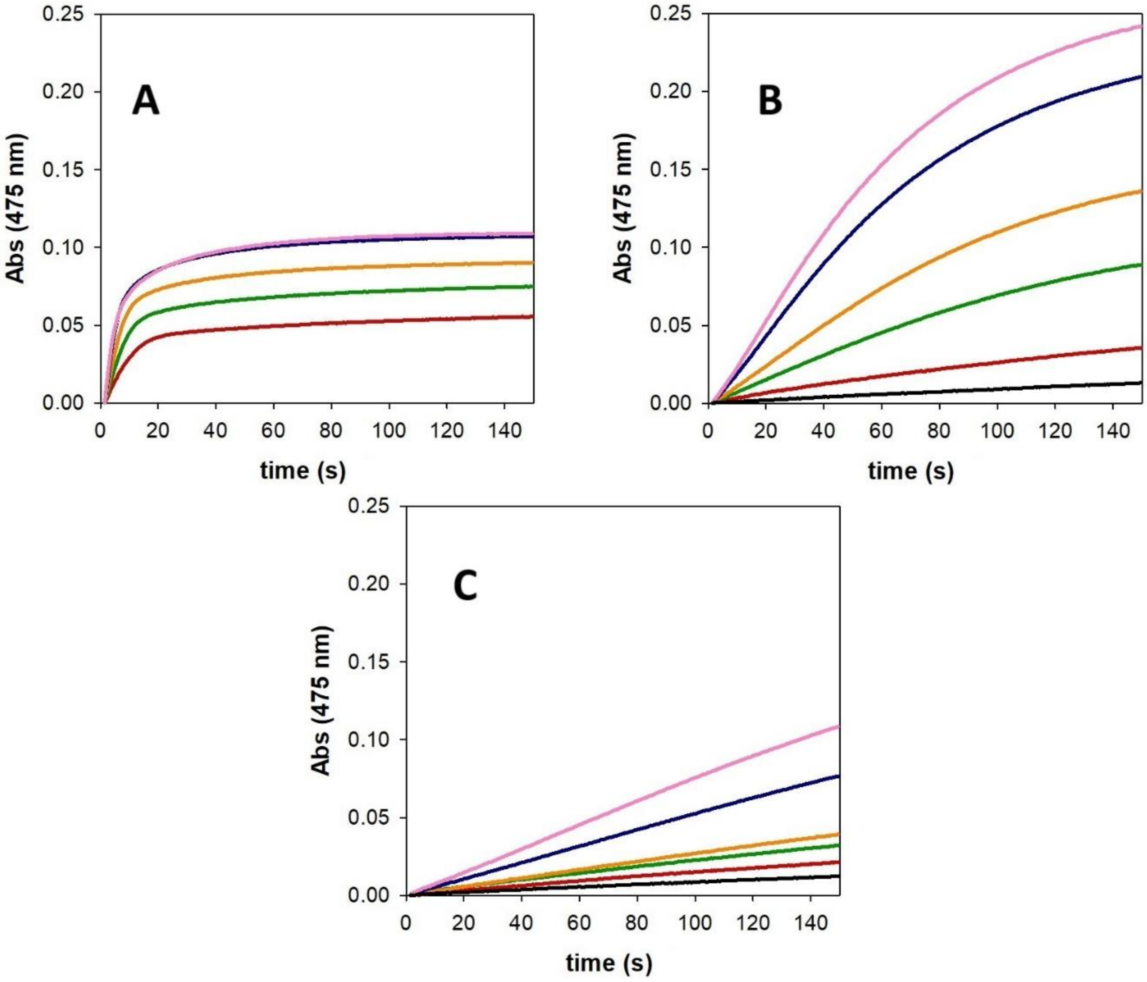

Figure S6. Kinetic profiles of DA (1 mM) oxidation in 20 mM phosphate buffer solution at pH 7.4 and 25 °C in the presence of hydrogen peroxide (100 mM, black traces) and hemin (0.2 μM, red traces) with increasing equivalents of Aβ(1-16) (4 μM, green; 10 μM, orange; 30 μM, blue; 50 μM, pink); 2 mM and 10 mM SDS (panels B and C, respectively) was added to the reaction media.

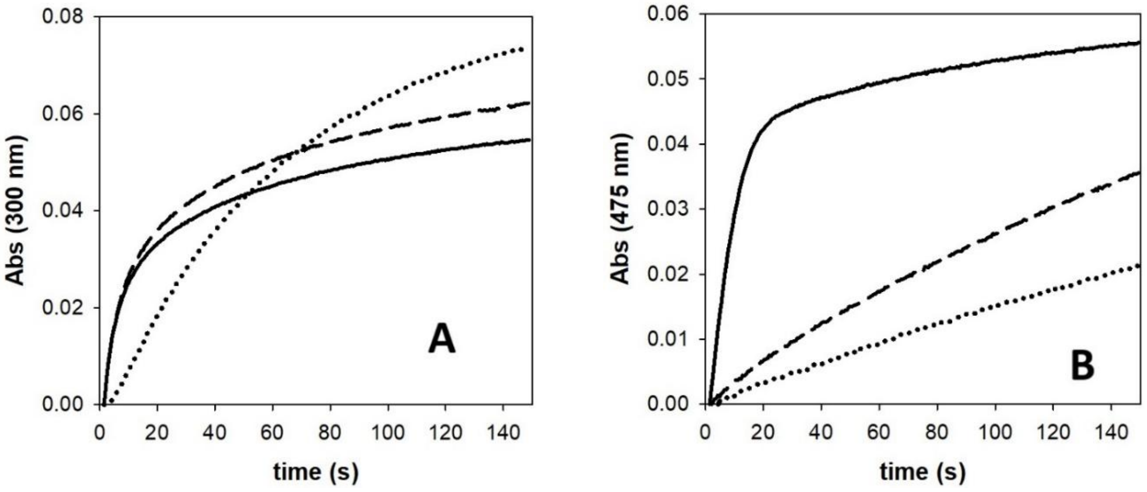

Figure S7. Kinetic profiles of HPA **(A)** and DA **(B)** (1 mM) oxidation in 20 mM phosphate buffer solution at pH 7.4 and 25 °C in the presence of hydrogen peroxide (40 mM, in **A** and 100 mM, in **B**) and hemin (2 μM, in **A** and 0.2 μM, in **B** – solid traces) with the addition of 2 mM (dashed) and 10 mM SDS (dotted).

Supporting information

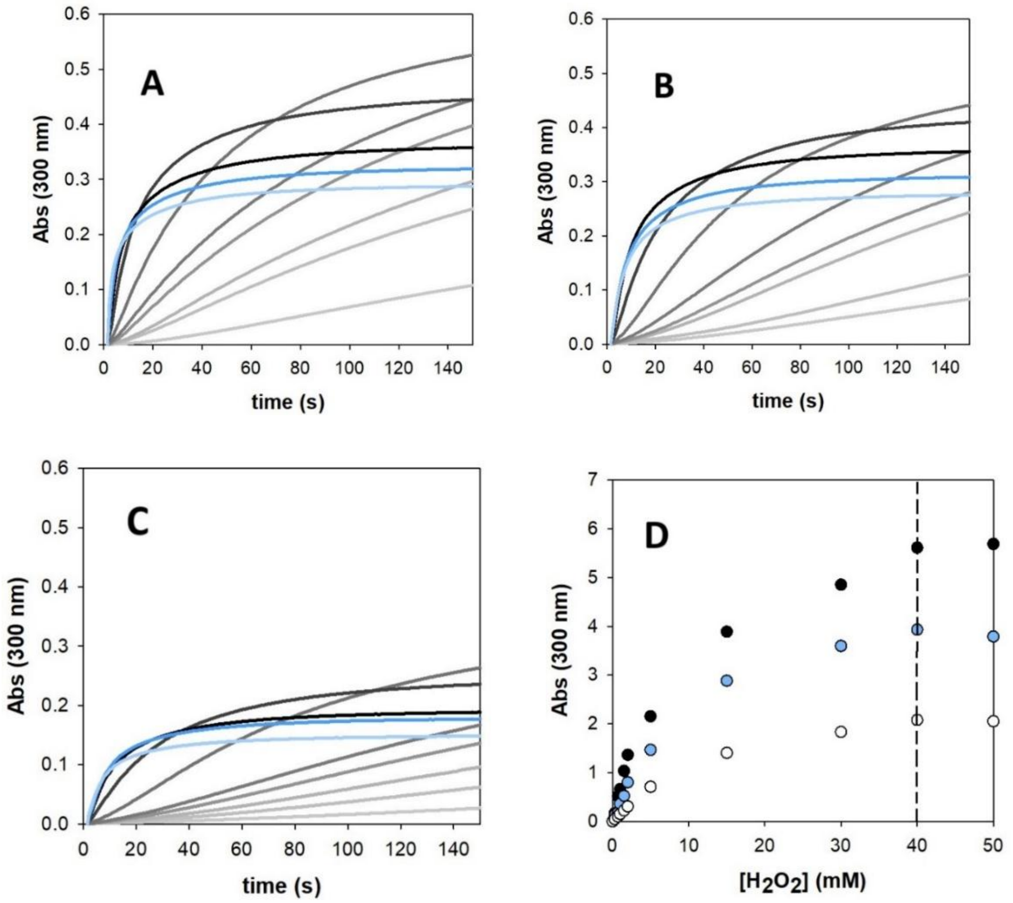

Figure S8. Kinetics of HPA (1 mM) oxidation in 20 mM phosphate buffer solution at pH 7.4 and 25 °C, performed in the presence of different amounts of H_2O_2 (0-50 mM), hemin (2 µM), Aβ(1-16) (30 µM - **A**) and SDS (**B** – 2 mM and **C**, 10 mM). Increasing black scale shows the data obtained with [H_2O_2] before the saturation value, while light blue traces are the kinetics obatined with over-saturating concentrations. **D)** Initial oxidation rates of HPA oxidation obtained from panels A (black circles), B (light blue) and C (white).

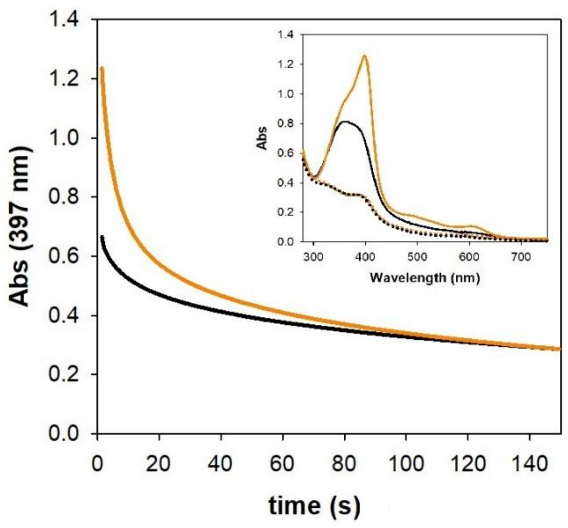

Figure S9. Kinetic profiles of hemin (2 µM) degradation monitoring the Soret band decrease at 397 nm in phosphate buffer solution (black trace) and in SDS micelle (10 mM - orange) promoted by the presence of hydrogen peroxide (100 mM); solid and dotted spectra show the Abs profiles of the hemin solutions at time 0 (after the addition of H_2O_2) and at the end of the kinetics, respectively.

Supporting information

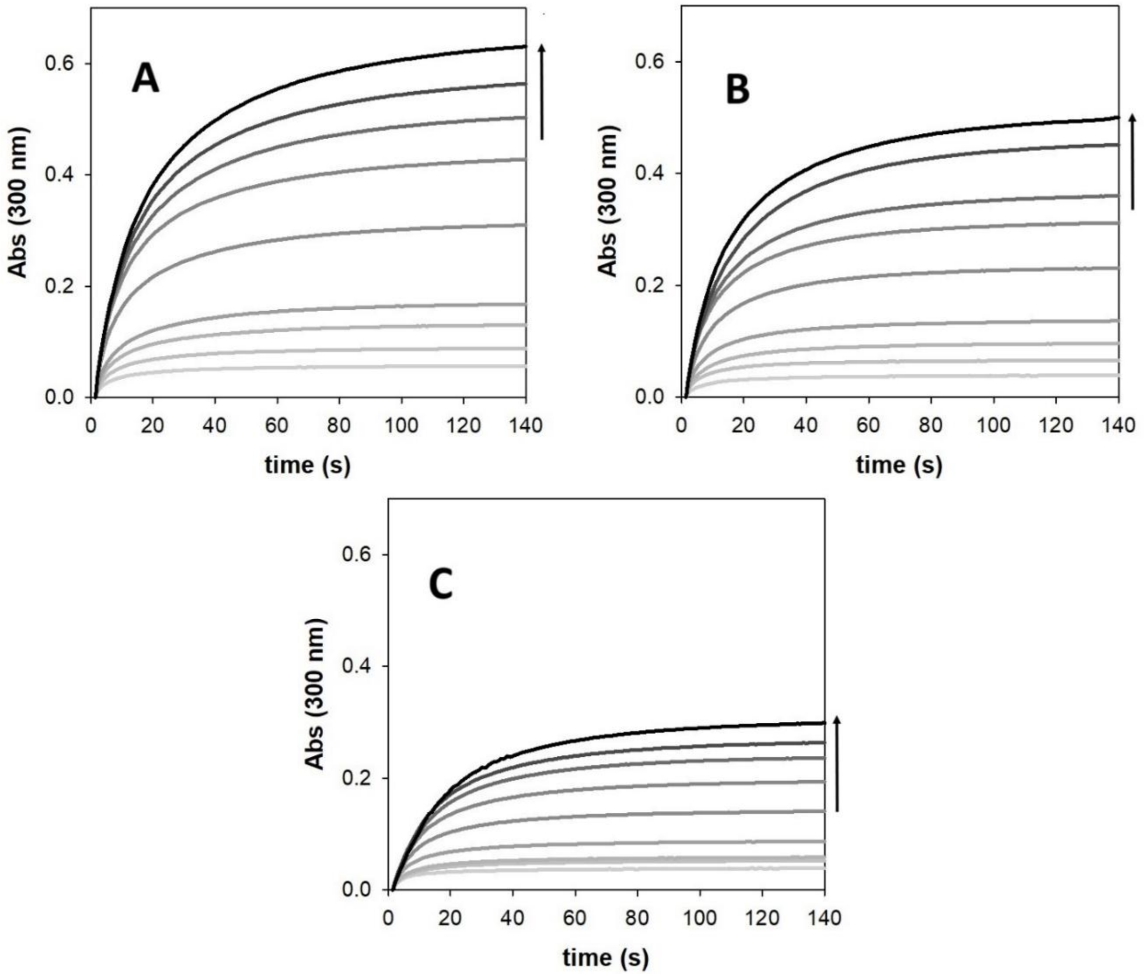

Figure S10. Kinetics of HPA oxidation performed at different concencentrations of substrate (0 - 4 mM) in the presence of hemin (2 μM) and Aβ(1-16) (30 μM – panel **A**), with the addition of SDS (**B** – 2 mM, and **C** – 10 mM).

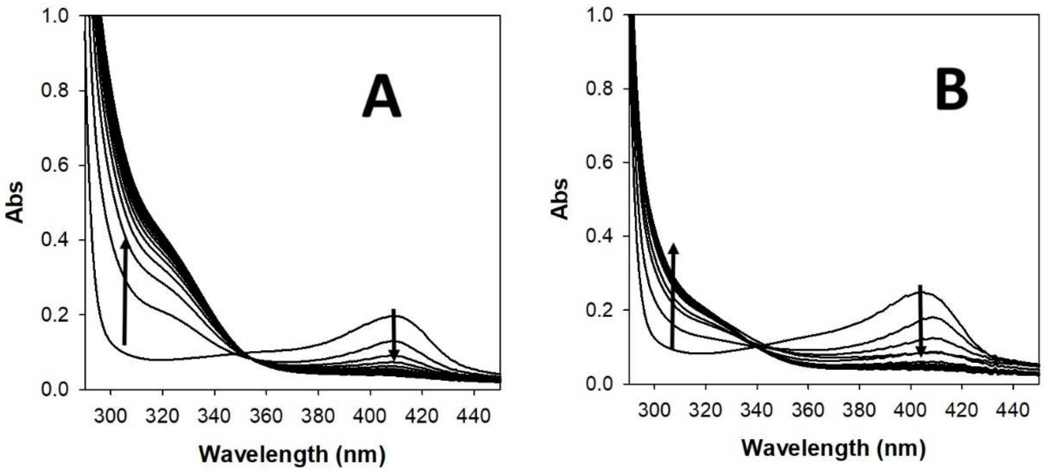

Figure S11. A) Selected absorption spectra of the oxidation reaction of HPA (4 mM) in phosphate buffer (20 mM), pH 7.4 and 25 °C, in the presence of hemin (2 μM), Aβ (30 μM), hydrogen peroxide (40 mM). **B)** Same conditions of the experiments in panel A) with addition of SDS 10 mM. at the following reaction times: 5 min (black dotted line), 20 min (black continuous line), 40 min (dark gray continuous line), 60 min (black dashed line), 100 min (light gray continuous line). For clarity we show only a selection of spectra (taken each 10 seconds), whereas the spectra were taken each 0.5 second for proper kinetic analysis.

Supporting information

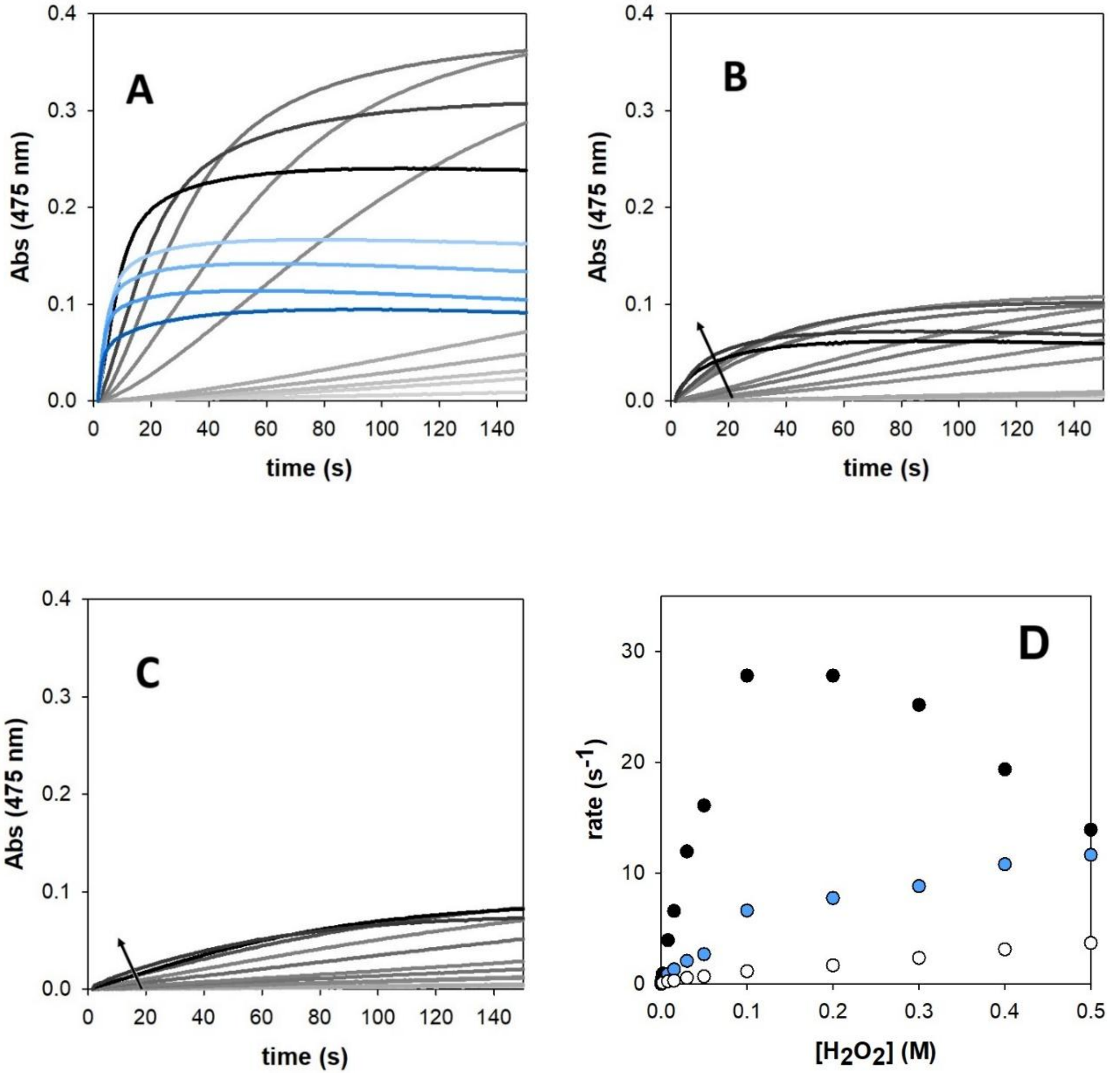

Figure S12. Kinetic profiles of oxidation of DA (1 mM), in 20 mM phosphate buffer solution at pH 7.4 and 25 °C, catalyzed by sub-/saturating amounts of H_2O_2 (0-500 mM), hemin (0.2 μM) and Aβ(1-16) (30 μM - **A**), with SDS (2 mM – **B**, and 10 mM – **C**). In panel A, increasing black scale shows the kinetics obtained with increasing amounts of hydrogen peroxide till the saturation value, while blue scale traces are the kinetic profiles obtained with over-saturating concentrations. **D)** Initial oxidation rates of the kinetic profiles shown in panel A (black circles), B (light blue) and C (white).

Supporting information

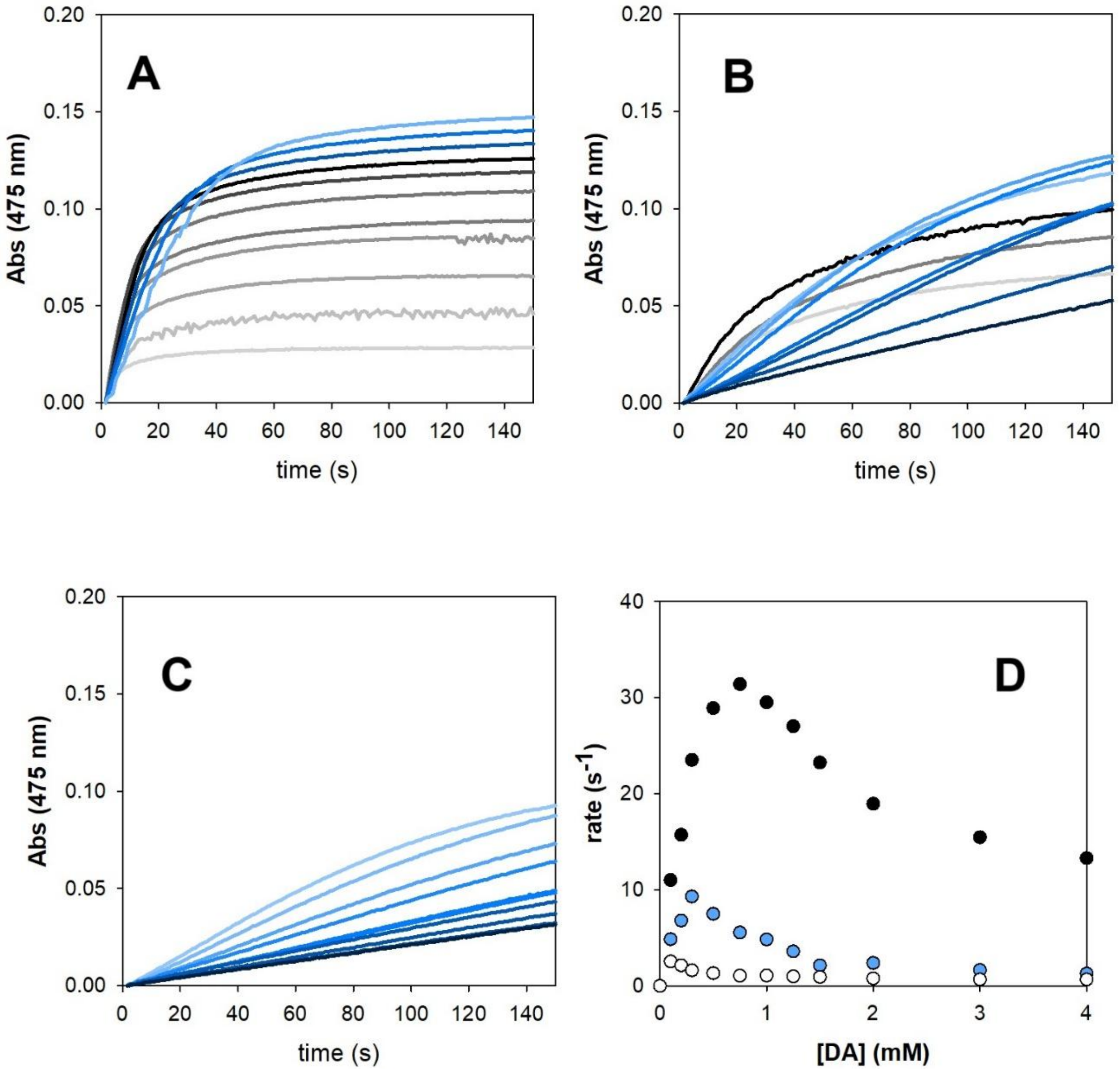

Figure S13. Kinetic profiles of oxidation of DA (0-4 mM), in 20 mM phosphate buffer solution at pH 7.4 and 25 °C, catalyzed by saturating amounts of H_2O_2 (100 mM), hemin (0.2 µM) and Aβ(1-16) (30 µM - **A**), with SDS (2 mM – **B**, and 10 mM – **C**). Increasing black scale (from light grey to black) shows the kinetics obtained with increasing amounts of substrate up to the point of maximal initial rate, whereas blue scale (from blue to light blue) shows the kinetic profiles obtained at the highest concentrations of substrate. **D)** Initial oxidation rates of the kinetic profiles shown in panel A (black circles), B (light blue) and C (white).

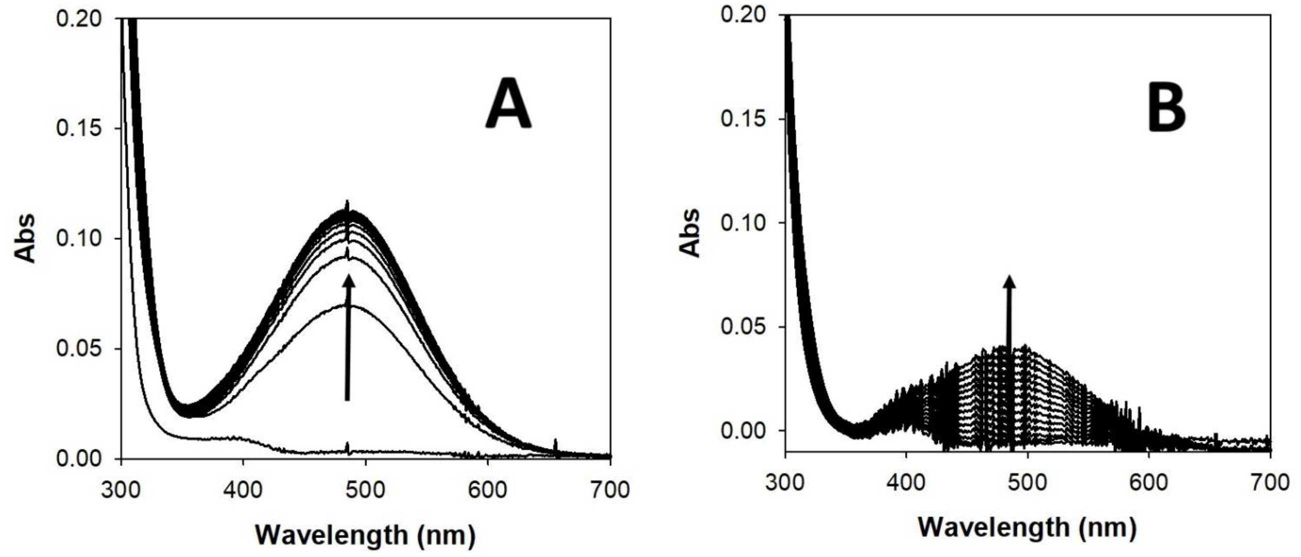

Figure S14. A) Selected absorption spectra of the oxidation reaction of DA (1 mM) in phosphate buffer (20 mM), pH 7.4 and 25 °C, in the presence of hemin (0.2 μM), Aβ (30 μM), hydrogen peroxide (100 mM). **B)** Same conditions of the experiments in panel A) with addition of SDS 10 mM. at the following reaction times: 5 min (black dotted line), 20 min (black continuous line), 40 min (dark gray continuous line), 60 min (black dashed line), 100 min (light gray continuous line). For clarity we show only a selection of spectra (taken each 10 seconds), whereas the spectra were taken each 0.5 second for proper kinetic analysis.

References

[47] C. Bacchella, S. De Caro, S. Nicolis, E. Monzani, S. Dell'Acqua, "Hemin, copper and amyloid-β: A medley involved in Alzheimer's disease. An interaction that fine regulates the reactivity". *J Inorg Biochem.* **2025**, 263: 112775.
[48] S.B. Brown, I.R. Lantzke, "Solution structures of ferrihaem in some dipolar aprotic solvents and their binary aqueous mixtures". *Biochem J.* **1969**, 115(2): 279-285.
[54] J.R. Ros, J.N. Rodríguez-López, F. García-Cánovas, "Tyrosinase: kinetic analysis of the transient phase and the steady state". *Biochim Biophys Acta.* **1994**, 1204(1): 33-42.
[59] V. Pirota, S. Dell'Acqua, E. Monzani, S. Nicolis, L. Casella, "Copper-Aβ Peptides and Oxidation of Catecholic Substrates: Reactivity and Endogenous Peptide Damage". *Chemistry*. **2016**, 22(47): 16964-16973.
[60] C. Redaelli, E. Monzani, L. Santagostini, L. Casella, A.M. Sanangelantoni, R. Pierattelli, L. Banci, "Characterization and peroxidase activity of a myoglobin mutant containing a distal arginine". *Chembiochem.* **2002**, 3(2-3): 226-233.
